# The effect of androgen receptor expression on clinical characterization of metastatic breast cancer

**DOI:** 10.18632/oncotarget.14414

**Published:** 2017-01-02

**Authors:** Ji-Yeon Kim, Kyunghee Park, Eunjin Lee, Hae Hyun Jung, Jin Seok Ahn, Young-Hyuck Im, Woong-Yang Park, Yeon Hee Park

**Affiliations:** ^1^ Division of Hematology-Oncology, Department of Medicine, Samsung Medical Center, Seoul 06351, Korea; ^2^ Samsung Genome Institute, Samsung Medical Center, Seoul 06351, Korea; ^3^ Biomedical Research Institute, Samsung Medical Center, Seoul 06351, Korea; ^4^ Department of Molecular Cell Biology, Sungkyunkwan University School of Medicine, Seoul 06351, Korea; ^5^ Samsung Advanced Institute for Health Sciences and Technology, Sungkyunkwan University School of Medicine, Seoul 06351, Korea

**Keywords:** androgen receptor (AR), metastatic breast cancer, RNA-Seq, prognosis

## Abstract

In breast cancer (BC), androgen receptor (AR) expression is related to estrogen receptor (ER) and/or progesterone receptor (PgR) expression. AR expression is an indicator of good prognosis in breast cancer regardless of hormone receptor (HR) status. In this study, we evaluated the effect of AR-related gene expression on clinical characterization of metastatic BC. We performed RNA-Seq to evaluate gene expression using mRNA extracted from 37 patients with metastatic BC. Intrinsic subtype prediction, analysis of differential gene expression, and gene set enrichment pathway analysis were then performed. Metastatic BCs were categorized into three subgroups based on AR, ER, PgR, and HER2 expression. According to this subcategorization, 70 genes including AR, ER, and HER2 were differentially expressed among the three groups. In gene set enrichment pathway analysis, the low AR group was associated with the cell cycle pathway, whereas mammalian target of rapamycin (mTOR) pathways was prevalent in the high ER and AR group. In survival analysis, a higher level of AR expression correlated with prolonged overall survival in metastatic BC (high expression vs. low expression, median OS 53.1 vs. 27.2 months, p=.001). In conclusion, we propose that AR and AR-related gene expression could be utilized to predict the prognosis of metastatic BC and thus may be useful in treatment planning for refractory BC.

## INTRODUCTION

Breast cancer (BC) consists of heterogeneous subtypes distinguished by different gene expression patterns [1]. Using mRNA expression array data, BC has been divided into five intrinsic subtypes: luminal A, luminal B, HER2 overexpression, normal breast-like and basal epithelial cell associated [2, 3]. However, the utility of microarray-based classification is limited due to the high cost and low accessibility. Instead, BC is usually classified into three subtypes according to estrogen receptor (ER)/progesterone receptor (PgR) expression and HER2 overexpression [4] using immunohistochemistry (IHC) on three surrogate markers. These three IHC molecular markers are used as reliable predictive and prognostic indicators for treatment planning in BC patients [5]. Hormone receptor (HR)-positive BC, which expresses ER and/or PgR, is sensitive to anti-estrogen treatment [6] and HER2 overexpression is a predictive marker of HER2-targeted treatment in HER2-positive breast cancer [7]. In contrast, triple-negative breast cancer (TNBC), BC without ER/PgR expression or HER2 overexpression, has heterogeneous histologic characteristics, no identified therapeutic target molecules [8], and a dismal prognosis [9].

In an effort to identify additional predictive biomarkers, many previous studies have investigated the androgen receptor (AR). AR is a member of the sex hormone receptor family, together with ER and PgR, and has been well studied in prostate cancer [10]. Testosterone and 5α-dihyroxyltestosterone stimulate AR in prostate cancer and facilitate cancer cell growth. Therefore, androgen-deprivation therapy is a standard treatment in prostate cancer [11]. In breast cancer, the level of AR expression is positively correlated with ER and/or PgR expression, and high AR expression is an indicator of good prognosis in HR-positive breast cancer [12]. In HR-negative BC, AR expression is more complex. Recent report suggested that HR-negative BC was categorized based on AR pathway activation and AR-activated HR-negative breast cancer showed a proliferative response to androgens without ER dependence [13]. In addition, TNBC could be divided into six categories, including the luminal AR (LAR) type [14] and this subtype is HR-negative, but gene ontologies are strongly enriched for steroid synthesis and estrogen/androgen metabolism. On the basis of AR expression in breast cancer, a phase II study of the AR inhibitor enzalutamide in TNBC with AR expression is now in progress [15]. Moreover, some research showed that AR expression might be a poor prognostic marker in TNBC and tamoxifen treatment could overcome survival disadvantage [16, 17]. In contrast, non-basal TNBC with AR expression might have good prognosis [18]. However, studies have yet to explore the relationship between AR expression and prognosis in metastatic BC.

In this study, we evaluated the activation of the AR pathway using RNA expression profiles as a biomarker in metastatic BC.

## RESULTS

### Clinical characteristics of metastatic breast cancer

We enrolled 54 patients with metastatic BC. Of these 54 patients, RNA sequencing was performed on 37 patients. Seventeen patients could not undergo RNA-Seq because of RNA extraction failure.

The demographic and clinical features of the 37 patients are summarized in Table 1. The median age of enrolled patients was 45.1 years. TNBC was 35.1% (13 patients) and basal-like BC subtype, according to intrinsic subtype, was 37.8% (14 of 37 patients). Testing for the *BRCA1/2* mutation was performed in five patients, and a germline *BRCA1* and/or *BRCA2* mutation was detected in three patients. Visceral metastasis was found in 15 patients; 8 patients had brain metastasis and the others had liver metastasis. On average, patients with metastatic breast cancer received more than three chemotherapeutic agents for palliative treatment (3.42 in ER+HER2- patients, 4.40 in ER+HER2+, 2.54 in ER-HER2- and 3.43 in ER-HER2+). Thirty-six of 37 patients received anthracycline-containing cytotoxic chemotherapy and 31 were treated with taxane chemotherapy. All patients with ER-positive breast cancer were treated with tamoxifen and/or non-steroidal aromatase inhibitor. Anti-HER2 treatment was administered to all patients with HER2-positive breast cancer.

**Table 1:** Clinicopathological characteristics of metastatic breast cancer (N=37)

	*N* = 37(%)
Age (median)	45.1±11.0
Range	26.5-75.7
<40 years old	15 (40.5)
≥40 years old	22 (59.5)
Histology	
Invasive ductal carcinoma	34 (91.9)
Other	3 (8.1)
Subtype	
ER+HER2-	12 (32.4)
ER+HER2+	5 (13.5)
ER-HER2-	13 (35.1)
ER-HER2+	7 (18.9)
Intrinsic subtype	
Luminal A	7 (18.9)
Luminal B	6 (16.2)
Basal-like	14 (37.8)
Normal-like	2 (5.4)
HER2-enriched	8 (21.6)
BRCA1/2	
Wild type	2 (5.4)
Mutated	3 (8.1)
Not tested	32 (86.5)
Cancer status	
Recurrent	27 (73.0)
Initially metastatic	10 (27.0)
Visceral metastasis	
Yes	15 (40.5)
Liver metastasis	7 (18.9)
Brain metastasis	8 (21.6)
No	22 (59.5)
Biopsy site	
Breast	12 (32.4)
Lymph node	7 (18.9)
Pleura	7 (18.9)
Liver	3 (8.1)
Lung	2 (5.4)
Other	6 (16.2)
Chemotherapy agents (average 3.24)	
1	8 (21.6)
2	11 (29.7)
3	4 (10.8)
≥4	14 (37.8)
Chemotherapeutic regimen	
Anthracycline	36 (97.3)
Taxane	31 (83.8)
Both anthracycline and taxane	27 (73.0)
Hormone therapy (N=17)	
Yes	17 (100.0)
No	0 (0.0)
HER2-targeted therapy (N=12)	
Yes	12 (100.0)
No	0 (0.0)

Time to RNA-Seq from diagnosis with metastatic breast cancer varied according to the subtype of breast cancer (Table 2). For ER-HER2+ breast cancer, mean time to RNA-Seq was 29.3 months (range 5.5-69.7) compared with 4.3 months (range 0.0-36.7) for ER-HER2- breast cancer.

**Table 2:** Previous chemotherapy and time to biopsy according to subtype

Subtype	No. of previous chemotherapy agents	Time to biopsy after metastasis
ER+HER2-	3.5 (range 1-6)	13.6 months (range 0.1-126.0)
ER+HER2+	4.4 (range 1-11)	18.8 months (range 2.4-33.2)
ER-HER2-	2.5 (range 1-6)	4.3 months (range 0.0-36.7)
ER-HER2+	3.4 (range 1-9)	29.3 months (range 5.5-69.7)

### Androgen receptor (AR) expression in metastatic breast cancer

We analyzed the influence of baseline characteristics on AR expression. Among many characters of BC, we found that AR expression level was associated with BC subtypes. TNBC expressed AR mRNA at a low level (p=.001) (Table 3), while high AR expression was observed in 66.7% of HR+HER2- BC, 80% of HR+HER2+ BC, and 71.4% of HR-HER2+ BC. Basal-like BC exhibited low AR mRNA expression compared to the other subtypes (p=.001). Other baseline characteristics including age, histology, *BRCA1/2* mutation status, visceral metastasis, and *de novo* BC did not affect the level of AR expression.

**Table 3:** Baseline characteristics according to AR expression (N=37)

	Low AR expressionN=20 (%)	High AR expressionN=17 (%)	p-value
Age (median)			.942
Range			
<40 years old	8 (40.0)	7 (41.2)	
≥40 years old	12 (60.0)	10 (58.8)	
Histology			.504
Invasive ductal carcinoma	19 (95.0)	15 (88.2)	
Other	1 (5.0)	2 (11.8)	
Subtype			.001
ER+HER2-	4 (20.0)	8 (47.1)	
ER+HER2+	1 (5.0)	4 (23.5)	
ER-HER2-	13 (65.0)	0 (0.0)	
ER-HER2+	2 (10.0)	5 (29.4)	
Intrinsic subtype			.001
Luminal A	3(15.0)	4 (23.5)	
Luminal B	0 (0.0)	6 (35.3)	
Basal-like	13 (65.0)	1 (2.7)	
Normal-like	1 (5.0)	1 (2.7)	
HER2-enriched	2 (10.0)	6 (35.3)	
BRCA1/2			.349
Wild type	2 (10.0)	0 (0.0)	
Mutated	2 (10.0)	1 (5.9)	
Not tested	16 (80.0)	16 (94.1)	
Cancer status			.080
Recurrent	16 (80.0)	9 (52.9)	
Initially metastatic	4 (20.0)	8 (47.1)	
Visceral metastasis			.942
Yes	8 (40.0)	7 (41.2)	
Liver metastasis	4 (50.0)	4 (57.1)	
Brain metastasis	4 (50.0)	3 (42.9)	
No	12 (60.0)	10 (58.8)	
PIK3CA (N=34)			.013
Mutated	2 (11.1)	8 (50.0)	
Wild type	16 (88.9)	8 (50.0)	
TP53 (N=34)			.071
Mutated	10 (55.6)	4 (25.0)	
Wild type	8 (44.4)	12 (75.0)	

We also analyzed the associations between somatic mutations and AR expression. PIK3CA and MUC16 mutation was frequently observed in high AR expressing breast cancer (p =.017and .013, respectively). EVC, GNAS, LRRIQ1, TRIO and TTN mutation were frequently detected in AR overexpressing BC in contrast ACACB and PCLO mutation were in low AR. However, there were no statistical significance. *TP53* was the most frequently mutated gene in all subtypes of metastatic BC (41.2%); however, *TP53* mutation was not related to AR expression (p=.182) (Table 3, Supplementary Table 1, and Supplementary Table 2).

An association between gene expression and AR expression was also detected. BC with high AR expression also exhibited high expression of the ER, AGR2, FOXA1 and GATA3 genes.

### Breast cancer categorization based on ER, PgR, HER2, and AR expression patterns

**Figure 1:**
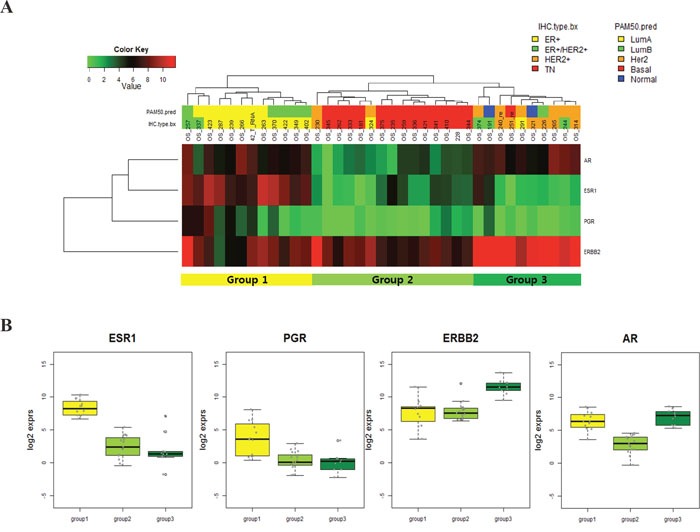
**A.** RNA expression profile of AR, ER, PgR and HER2 in metastatic BC; **B.** RNA expression profile of AR, ER, PgR and HER2 in metastatic BC according to subgroup.

We divided BC into three subgroups according to the expression profiles of ER, PgR, HER2, and AR (Figure 1A and Figure 1B). Group 1 had high ER and AR expression, whereas Group 3 had high HER2 and AR expression. Group 2 lacked AR, ER, and PgR expression. Compared with conventional subtype classification using ER, PgR and HER2 expression, the ER+HER2- and ER+HER2+ subtype were both included in Group 1; all TNBC subtypes, one ER+HER2- subtype, and one ER-HER2+ subtype were in Group 2; and the HER2+ subtype and one ER+HER2- subtype were in Group 3.

For validation of subcategorization according to four gene expression, we performed nCounter gene expression assay using same metastatic BC samples. Of total 37 samples, 30 samples were passed quality control and finally analyzed their RNA quantity.

This gene expression analysis showed the same result of metastatic BC sub-categorization as that from RNA-Seq data analysis (Supplementary Figure 1). There were three subgroups according to ER, PgR, HER2 and AR expression as like as RNA-Seq data analysis (Figure 1A). Moreover, all samples were divided into same sub-groups regardless of RNA expression detection technique.

**Figure 2:**
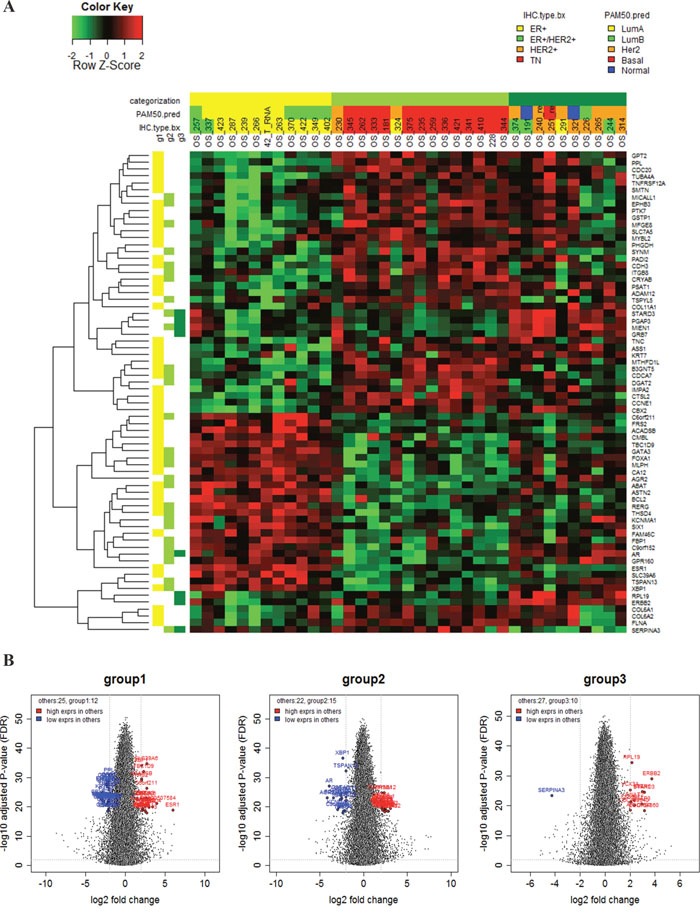
**A.** Seventy gene expression profiles according to subgroup; **B.** Volcano plots of differential gene expression according to subgroup.

Using this categorization, we found 70 differentially expressed genes (DEGs) to determine the characteristics of three groups (Figure 2A and Figure 2B). GATA3, FOXA1 and AGR2 upregulation was marked in Group 1, whereas high HER2, STARD3, GRB7 and AR expression was associated with Group 3. In Group 2, downregulation of ER, AR and HER2 and upregulation CDH3 and CCNE1 were observed. However, the PgR expression level did not vary among these three groups and PgR was not included in the 70 genes.

**Figure 3:**
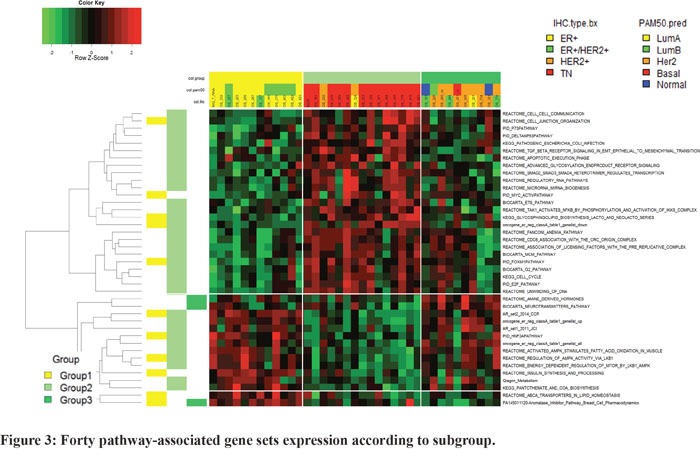
Forty pathway-associated gene sets expression according to subgroup

In pathway analysis, group-specific pathway analysis indicated that 40 pathway-associated gene sets were related to subcategorization (Figure 3). The upregulation of cell cycle-associated genes was observed in Group 2, and AR and mammalian target of rapamycin (MTOR) pathway genes were markedly upregulated in Group 1. Group3, representing high AR and HER2 expressing BC, neutrotransmitters pathway and amine derived hormones pathway were upregulated.

### The impact of AR expression on the prognosis of metastatic BC

**Figure 4:**
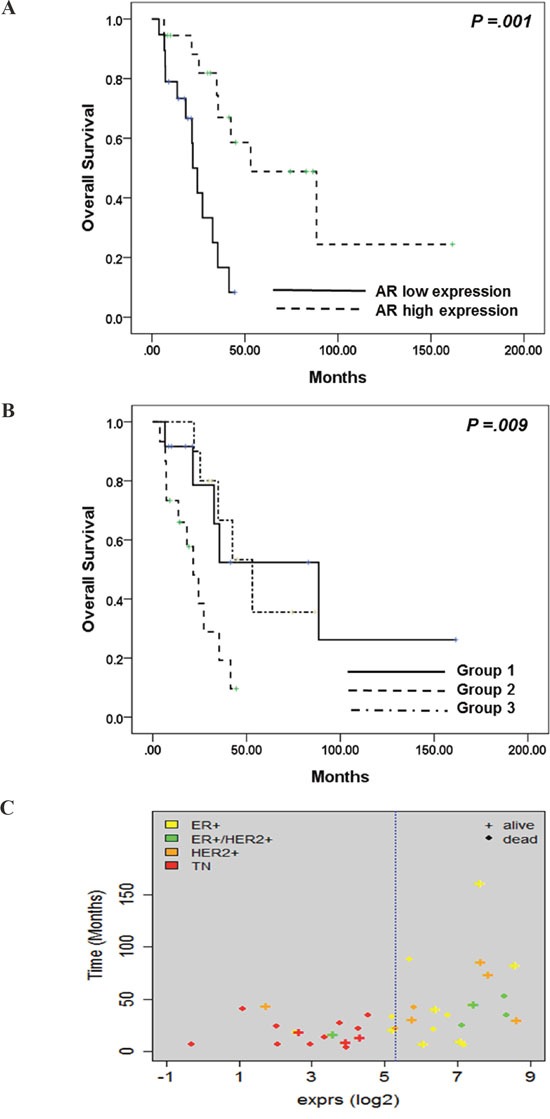
**A.** Kaplan-Meier analysis of overall survival according to AR expression; **B.** Kaplan-Meier analysis of overall survival according to AR, ER, PgR, and HER2 expression; **C.** Association between AR expression and IHC subtype.

We analyzed the association between AR expression and BC prognosis. AR expression was related to overall survival (high expression vs. low expression, median OS 53.1 vs. 27.2 months, p=.001) (Figure 4A). In addition, survival analysis suggested that the three groups categorized according to AR, ER, PgR, and HER2 expression exhibited differential survival (Figure 4B). Group 1 had the longest overall survival (Group 1 vs. 2 vs. 3: median OS 88.5 vs. 21.5 vs. 53.1 months, p=.009), whereas the survival duration of Group 2, the group with low expression of AR, ER, and PgR, was only one-fourth that of Group 1.

Survival duration from tumor biopsy was also affected by AR expression, but without statistical significance (p=.102) (Supplementary Figure 2A). There also seemed to be a correlation between survival duration and sub-categorization according to AR, ER, PgR, and HER2 expression (Supplementary Figure 2B).

### The collaboration of AR and PgR expression: simple way to determine BC prognosis

**Figure 5:**
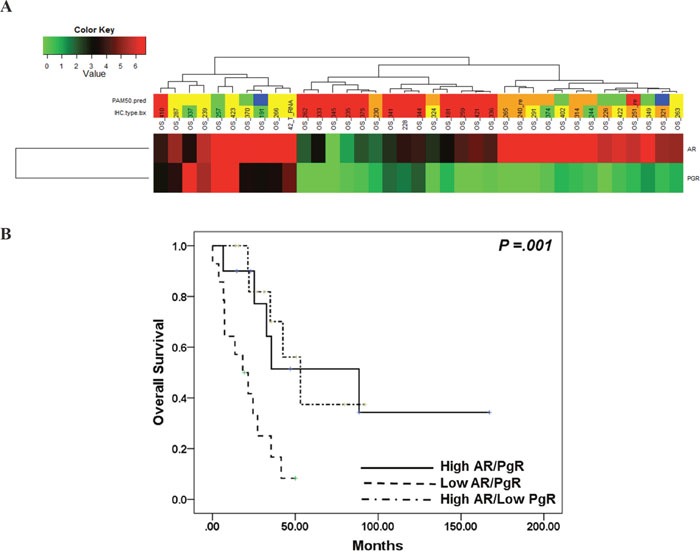
**A.** Sub-classification according to AR and PgR expression; **B.** Kaplan-Meier analysis of overall survival according to AR and PgR expression.

Further gene expression analysis was performed using two genes; AR and PgR. As like as previous analysis, this analysis divided into three subcategorization; high AR and PgR, high AR and low PgR and low AR/PgR expression. There was no BC with high PgR and low AR expression. Further survival analysis according to this categorization showed that the group with high AR expression had better survival outcome compared to that of low AR expression, regardless of PgR expression (High AR/PgR vs. low AR/PgR vs. high AR and low PgR: median overall survival 88.5 vs. 18.2 vs. 53.1 months, p=.001) (Figure 5A and 5B).

### Gene expression profile of the AR pathway

We focused on the gene sets of the AR pathway that were previously published and used in TNBC characterization (12,13,18) (Supplementary Table 2). These gene sets are called the luminal AR (LAR) and TNBC with LAR expression sets and are associated with a good prognosis.

First, we classified BC into two groups using the ER-negative, class A gene sets described by Doane et al.(12). In this analysis, 97 of 101 reference genes were included in the RNA-Seq results. Group 1 consisted of the TNBC subtype, except for one HER2+ BC. One TNBC was included in Group 2, which consisted of HR+ and/or HER2+ BC (Supplementary Figure 3). Thirty-two of 97 genes were upregulated in Group 1, and 65 genes, including AR, were overexpressed in Group 2. We were also able to classify BC according to the 15 genes described by Burstein et al.(18). In this analysis, 37 BCs were divided into two groups: one group expressed high AR, ER, RET, ERBB3, and ERBB4 and was mainly composed of non-TNBC cases, whereas the other group was mainly comprised of TNBCs (Supplementary Figure 4). In contrast to the above classifications, categorization according to the expression of 10 genes described by Lehmann et al.(13) yielded three different groups composed of heterogeneous subtypes of BC (Supplementary Figure 5).

## DISCUSSION

Here, we identified AR expression in metastatic BC. Although AR expression was dependent on ER/PgR and HER2 expression, our research suggests that AR expression has an additional role as a prognostic marker of metastatic BC. Moreover, the impact of AR expression seemed to override conventional BC subtypes based on ER, PgR, and HER2 expression upon subcategorization.

Previous research on AR expression in BC reported that AR expression was positively correlated with ER expression and only affected ER-positive breast cancer [12, 19]. AR expression was associated with better prognosis compared to AR-negative BC. However, these studies were conducted using specific subtypes of BC. In our study, AR expression was a good prognostic marker in BC, regardless of subtype. Additional analysis using only PgR and AR expression sufficed indicating BC prognosis. In this analysis, AR status was the most important factor predicting patient prognosis.

We divided subjects into three subgroups according to ER, PgR, HER2, and AR expression and performed different gene and pathway expression analyses with regard to these three subgroups. Interestingly, PgR expression did not differentiate the three groups and might not have a role in the characterization of metastatic breast cancer. This trend was consistently observed in immunohistochemistry, and PgR mRNA expression was positively correlated to the IHC profile of PgR in metastatic BC. Considering that our metastatic BC cohort was composed of highly refractory BCs and genetic loss of PgR gene copy/mRNA expression was frequently observed in ER-positive breast cancer with poor prognosis [20], our results suggest that the significance of PgR expression in refractory BCs decreased when AR expression was considered in the sub-categorization.

Moreover, these three subgroups exhibited enrichment of different pathway-associated gene sets, which might provide a treatment strategy for patients with refractory BC. ER and AR overexpressed BC had activated mTOR pathway signal. Previous research conducted in prostate cancer showed that AR positively regulated mTOR activity and compensatory increase of AR function due to a repressed mTOR signal is advantageous for tumor cell survival [21]. Therefore, combination of mTOR inhibitor and AR inhibitor would be the effective therapeutic strategy in this group of metastatic BC. In addition, group 2, represented by low ER and AR was activated in cell cycle signal and cytotoxic chemotherapy might be more effective.

Although systemic treatment strategies for metastatic BC have progressed significantly, metastatic BC remains a challenging disease to treat effectively. In current practice guidelines for BC [22], patients with metastatic BC are treated according to HR expression and HER2 overexpression/amplification. Recently, AR expression was studied in metastatic BC, and clinical trials in AR-positive TNBC patients are ongoing. Previous phase II clinical trials of bicalutamide and enzalutamide, non-steroidal anti-androgen agents, in AR-expressing TNBC suggested that anti-androgen treatment had a marginal clinical benefit and a tolerable toxicity profile [23]. In a phase II clinical trial of enzalutamide for metastatic TNBC, investigators searched for predictive markers of response to anti-androgen therapy and found that, while AR immunohistochemistry was not predictive, the expression profile of 521 genes in the AR pathway (PREDICT AR) could predict response to anti-androgen treatment [15]. We performed pathway analyses using previously reported gene sets such as PREDICT AR. As expected, these analyses categorized subjects into TNBC and non-TNBC subgroups. One TNBC patient was classified into the non-TNBC subgroup; we hypothesize that this case might have been of the luminal AR (LAR) type and that anti-androgen treatment would be effective against this TNBC.

Lastly, this categorization revealed differences in overall survival (p=.009). In particular, one patient with ER+ BC in Group 2 had a relatively short OS (17.4 months) for a patient with ER+ breast cancer. Therefore, AR expression might be an important biomarker in metastatic BC when added to the current molecular biomarkers of ER, PgR, and HER2.

We performed RNA-Seq on 37 metastatic breast cancer tissues to detect significant gene expression among all of the expressed genes. Using RNA-Seq, we found that the AR gene and pathway were significant factors in the characterization of metastatic BC. Small sample size was the limitation of this study causing insignificant statistical differentiation.

IHC is routinely used in clinics to detect ER, PgR and HER2 protein expression while RNA-Seq is not employed in general practice because of the high cost and low accessibility. However, considering that intrinsic BC subtype based on mRNA expression profile is one of the most important molecular markers of this disease [2, 3], RNASeq, which generates in-depth RNA expression data, could be an important tool with central role in BC treatment planning. Moreover, in terms of AR, IHC is not standardized and is not a predictive marker of anti-androgen treatment. Considering previous studies presented gene expression profile helps prediction of anti-androgen treatment, we might suggest the level of AR transcriptome implicated in path of metastatic BC.

Our explorative study evaluated the role of AR expression in refractory BC regardless of subtype. Characterization using expression levels of AR and AR-related genes might benefit treatment planning for refractory BC and enable accurate prognosis prediction for metastatic BC. Further studies with a large sample size are warranted to validate our findings. Furthermore, anti-androgen therapy could be a new treatment strategy for patients suffering from refractory BC.

## MATERIALS AND METHODS

### Patients

This study was conducted as a prospective explorative analysis of patients with metastatic breast cancer at Samsung Medical Center. Women diagnosed with stage IV BC or recurrent BC after curative treatment via diagnostic examination and a staging work-up (breast magnetic resonance imaging [MRI], chest computed tomography [CT] scan, abdominal CT scan, bone scan, and/or positron emission tomography [PET]-CT scans if indicated) were included.

All patients provided written informed consent, and study approval was obtained from the Institutional Review Board of Samsung Medical Center, Seoul, Korea (IRB No: SMC 2012-08-065).

### IHC staining

Two experienced pathologists reviewed all pathology specimens to determine IHC staining for ER, PgR, and HER2. ER and PgR positivity were defined using Allred scores ranging from 3 to 8 based on IHC using antibodies to ER (Immunotech, Marseille, France) and PgR (Novocastra Laboratories Ltd., Newcastle upon Tyne, UK). HER2 status was evaluated using a specific antibody (Dako, Glostrop, Denmark) and/or silver *in situ* hybridization (SISH). Grades 0 and 1 for HER2, as assessed by IHC, were defined as a negative result, and grade 3 was defined as a positive result. Amplification of HER2 rated as 2+ by IHC was confirmed by SISH. Triple negativity was defined as a lack of expression of ER, PgR, and HER2.

### RNA extraction

Areas containing representative invasive breast carcinoma were outlined on the slide. Total RNA was then extracted using a High Pure RNA Paraffin kit (Roche Diagnostic, Mannheim, Germany) and the RNA concentration and 260/280 and 260/230 nm ratios were measured using a NanoDrop ND-1000 Spectrophotometer (NanoDrop Technologies, Rockland, DE, USA). Samples with less than 1 μg/μL total RNA even after concentration with a SpeedVacTM concentrator (Thermo Scientific™, Waltham, MA, USA) were excluded from downstream analysis.

### RNA-Seq analysis and normalization

After trimming the poor-quality bases from FASTQ files for whole transcriptome sequencing, the reads were aligned to the human reference genome hg19 with Tophat (version 2.0.6) and reference-guided assembly of transcripts with Cufflinks (version 2.1.1) was performed. The alignment quality was verified with SAMtools (version 0.1.19). Transcript abundance was estimated using a count-based method with htseq-count. Gene counts were used as input for TMM (Trimmed Mean of M values) normalization of the R package edgeR [24], and normalized counts were transformed to log2-counts per million (logCPM) by applying voom from the R package limma [25] to account for higher variability at low expression levels. Genes with zero read counts across all samples were removed for a more powerful statistical test (Supplementary Figure 6).

### Intrinsic subtyping

We performed intrinsic subtyping with log-scaled normalized expression values using the 50-gene Prediction Analysis of Microarray (PAM50) subtype predictor as described by Parker et al.[26]. The PAM50 subtype predictor classified tumors into the following groups: Luminal A, Luminal B, HER2-enriched, basal-like, and normal-like (Supplementary Figure 6).

### Survival analysis

We evaluated the association between gene expression and overall survival (OS) using the R package (Supplementery Figure 7). OS was defined as the elapsed time between the date of stage IV breast cancer diagnosis and the date of death. For each gene, patients were grouped based on the normalized expression value of the gene, with the top 50% and the bottom 50% representing high and low expression groups, respectively. Survival curves for the two groups were estimated with the Kaplan-Meier method, and the log-rank test was used to compare overall survival curves between the two groups (p<0.05). Fisher's exact test was used to identify the pathways that were enriched with significant associated genes in terms of overall survival (p<0.05).

### Gene set enrichment analysis

To examine how overall survival-associated genes share predefined gene sets representing common processes, pathways, and underlying biological themes, we investigated sub-collections in the Molecular Signatures Database (MSigDB, version 5.0) with OS-associated genes using the Gene Set Enrichment Analysis website. We also calculated Gene Set Enrichment scores for canonical pathways in MSigDB and several AR-related gene sets from the literature [13, 27] using the R package Gene Set Variation Analysis (GSVA) (Supplementary Figure 8). GSVA is a nonparametric method that provides sample-wise gene set enrichment scores to identify differential gene set activity. A two-sample t-test was then performed, and gene sets with a false discovery rate (FDR) less than 0.05 were considered to show significant differential activity between the two groups. All normalization, statistical analyses, and visualization were conducted within the R statistical system (version 3.0.2).

### DNA extraction

Tumors consisting of >75% malignant cells were dissected under a microscope from 4-mm unstained sections in comparison to an H&E-stained slide, and genomic DNA was extracted using a Qiagen DNA FFPE Tissue kit (Qiagen, Hilden, Germany) according to the manufacturer's instructions. After extraction, concentration as well as 260/280- and 260/230-nm ratios were measured by spectrophotometry (ND1000, NanoDrop Technologies, ThermoFisher Scientific, MA, USA). Each sample was then quantified using a Qubit fluorometer (Life Technologies, Carlsbad, CA, USA). Genomic DNA with a total yield >10 ng was used for library preparation.

### Whole exome sequencing

Poor quality reads were filtered out and aligned to the human reference genome (hg19) using Burrows-Wheeler Alignment tool (BWA, version 0.7.5a). In order to convert Sequence Alignment and Mapping (SAM) files into Binary Alignment and Mapping files (BAM) we used SAMtools (version 0.1.19). Polymerase chain reaction (PCR) duplicates were removed from the BAM files by Picard (version 1.93,http://picard.sourceforge.net/) and SAMtools before variant calling. The Genome Analysis Toolkit (GATK, version 2.4.7) was used to recalibrate base quality and optimizing local realignment. Single nucleotide variants (SNVs) and indels were called using muTect (version 1.1.4) and Varscan2 (version 2.3.5) by default parameter settings. Copy number variations were detected using CONTRA (version 2.0.4). Variants were annotated using ANNOVAR, with gene, chromosomal information, exonic function function (synonymous, nonsynonymous, stop gain, nonframeshift or frameshift indel), amino acid change, allele frequency in frequency in public databases such as 1000 Genomes Project (2012 February version) and dbSNP version (version 132, 137).

Variants that were located in the exonic regions with sufficient coverage (minimum depth of coverage ≥8) and variant allele frequency (VAF ≥0.1) were chosen for further statistical analyses. Synonymous variants were filtered out. Read alignments were manually investigated using the Integrative Genomic Viewer (http://www.broadinstitute.org/igv/)

Fisher's exact test was used for the analysis of mutations and polymorphic variants separately, to discover variants that were enriched in the patients with a favorable outcome. P-values <0.05 were considered significantly different. All statistical analyses, plots and heatmaps were conducted using R version 3.0.2 (http://www.R-project.org/).

## SUPPLEMENTARY MATERIALS FIGURES AND TABLES


