# Supplementary material for: The effect of androgen receptor expression on clinical characterization of metastatic breast cancer
Source: Oncotarget. 2017 Jan 2;8(5):8693–706. doi: 10.18632/oncotarget.14414 (PMC5352433; doi:10.18632/oncotarget.14414)
Supplement: Supplementary file 1 [file oncotarget-08-8693-s001.pdf]

The effect of androgen receptor expression on clinical characterization of metastatic breast cancer

SUPPLEMENTARY FIGURES AND TABLES

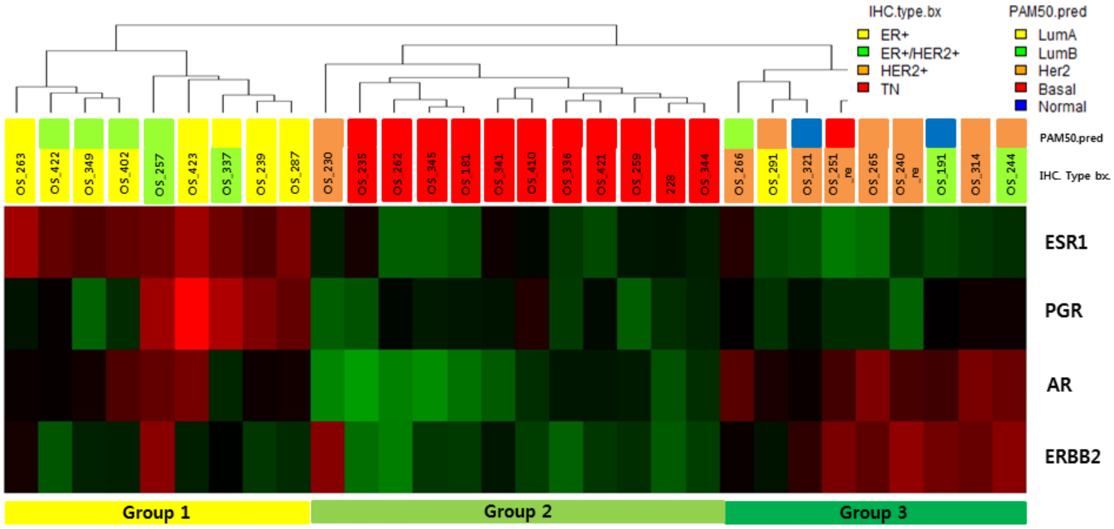

Supplementary Figure 1: nCounter gene expression assay of AR, ER, PgR and HER2 in metastatic BC.

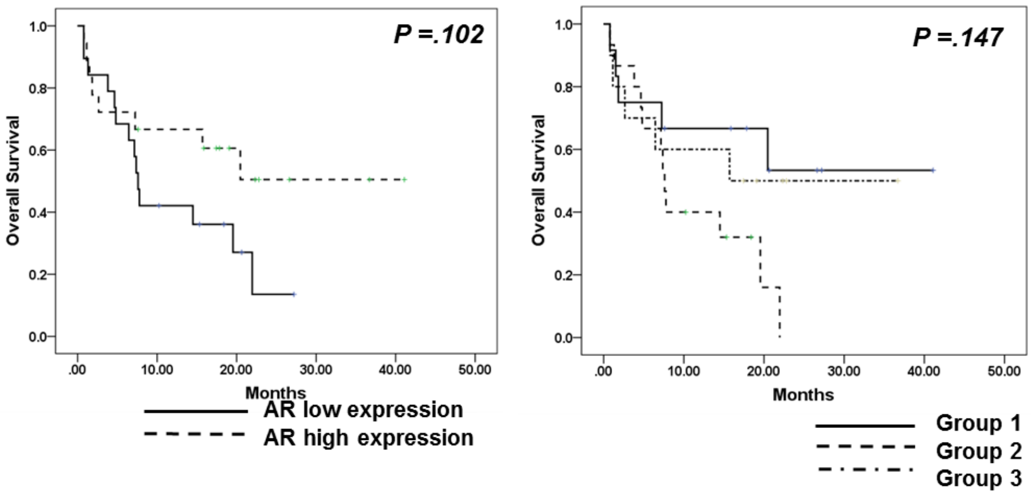

Supplementary Figure 2: A. Kaplan-Meier analysis of overall survival from biopsy according to AR expression; **B.** Kaplan-Meier analysis of overall survival from biopsy according to AR, ER, PgR and HER2 expression.

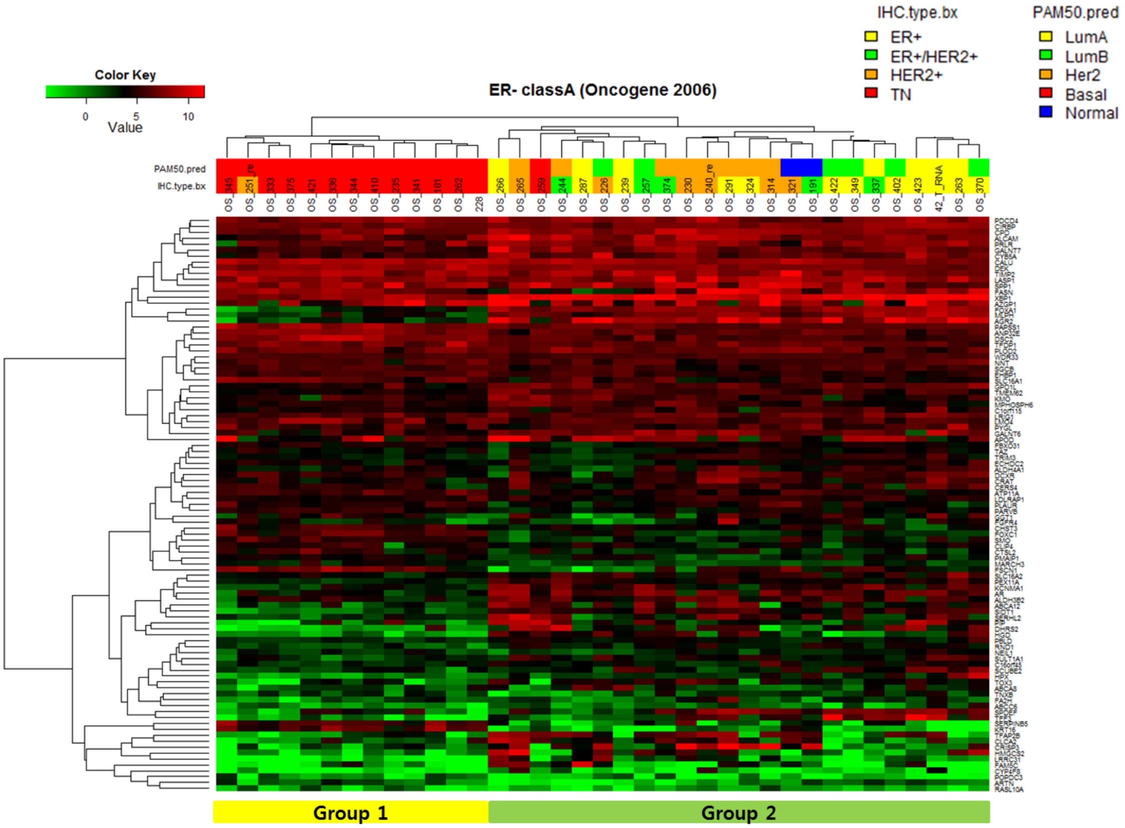

Supplementary Figure 3: Refractory BC classification using ER-Class A genes (Oncogene 2006).

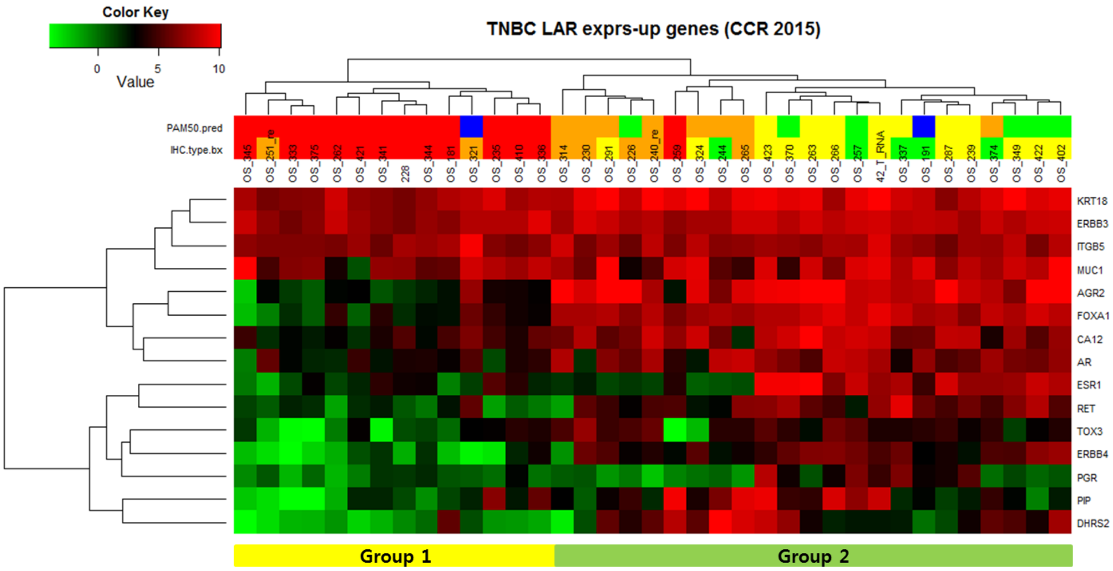

Supplementary Figure 4: Refractory BC classification using TNBC LAR genes (CCR 2015).

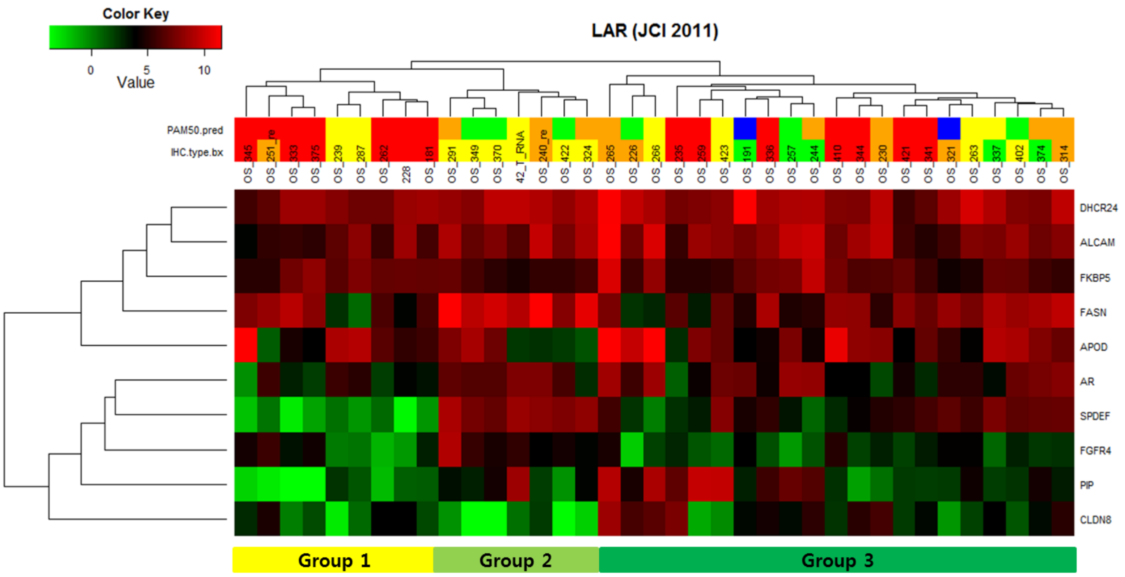

Supplementary Figure 5: Refractory BC classification using TNBC LAR genes (JCI 2011).

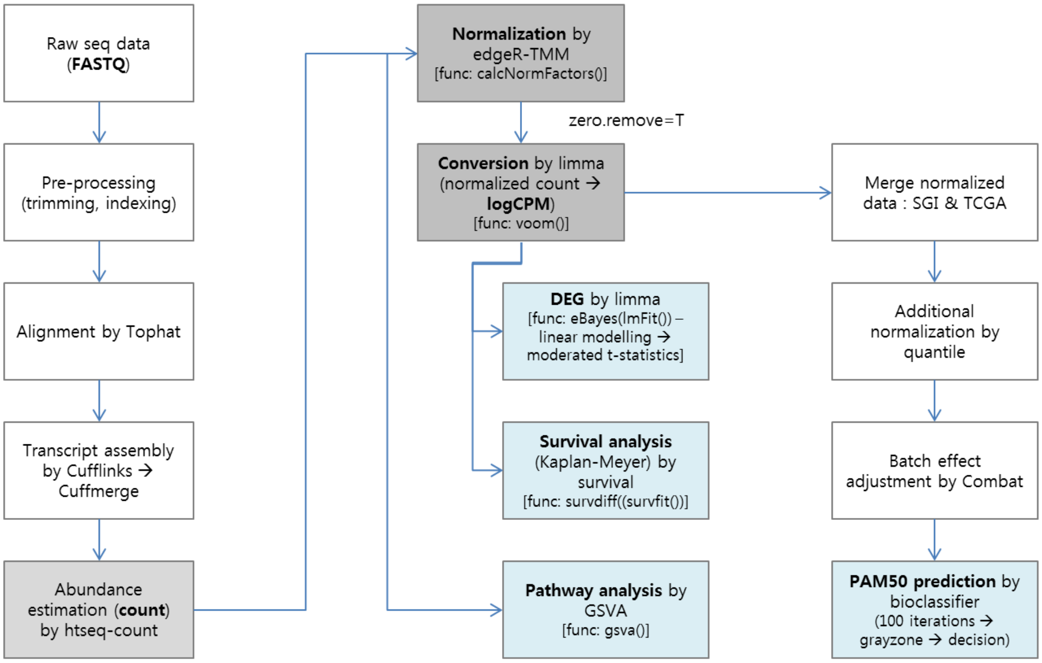

- Pipeline: PLoS Comput Biol 11(8): e1004393 (2015)
- Method comparison: Brief Bioinform. 2015 Jan;16(1):59-70
- limma for RNA-seq: Nucleic Acids Res. 2015 Apr 20;43(7):e47, Genome Biol. 2014 Feb 3;15(2):R29
- **log2-counts per million (logCPM)** with associated precision weights, by definition normalized for sequencing depth
- GSVA for RNA-seq: BMC Bioinformatics. 2013 Jan 16;14:7

Supplementary Figure 6: Pipeline of RNA-Seq data analysis.

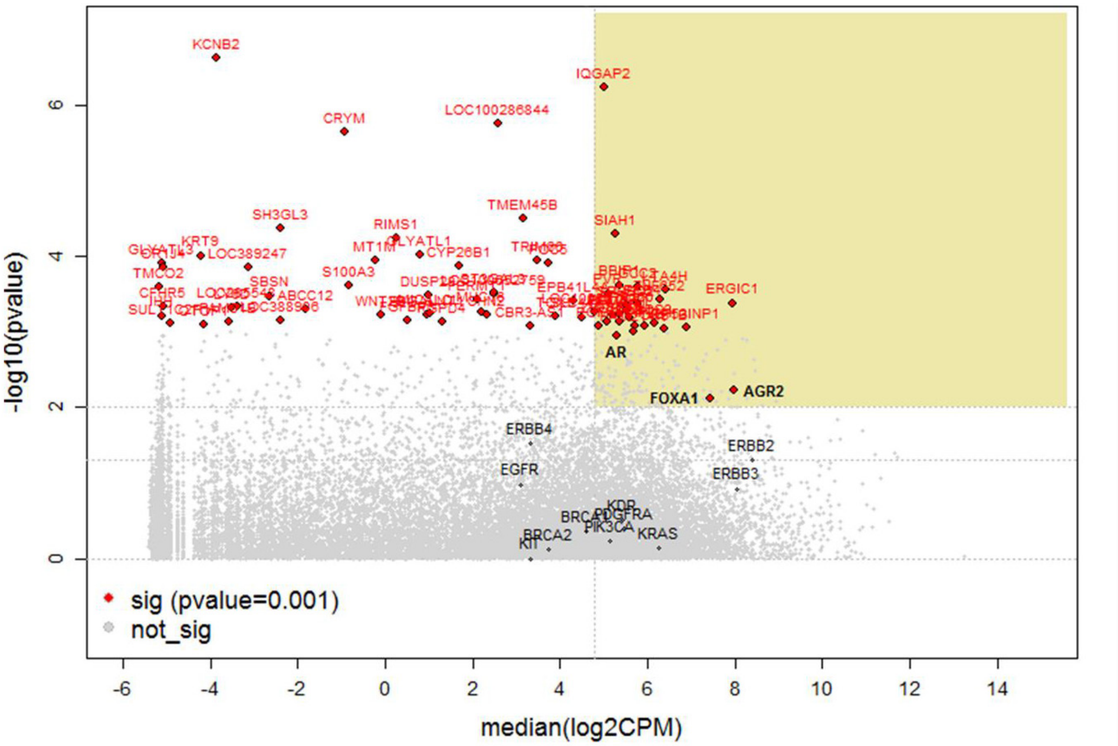

Supplementary Figure 7: Gene expression associated with BC prognosis.

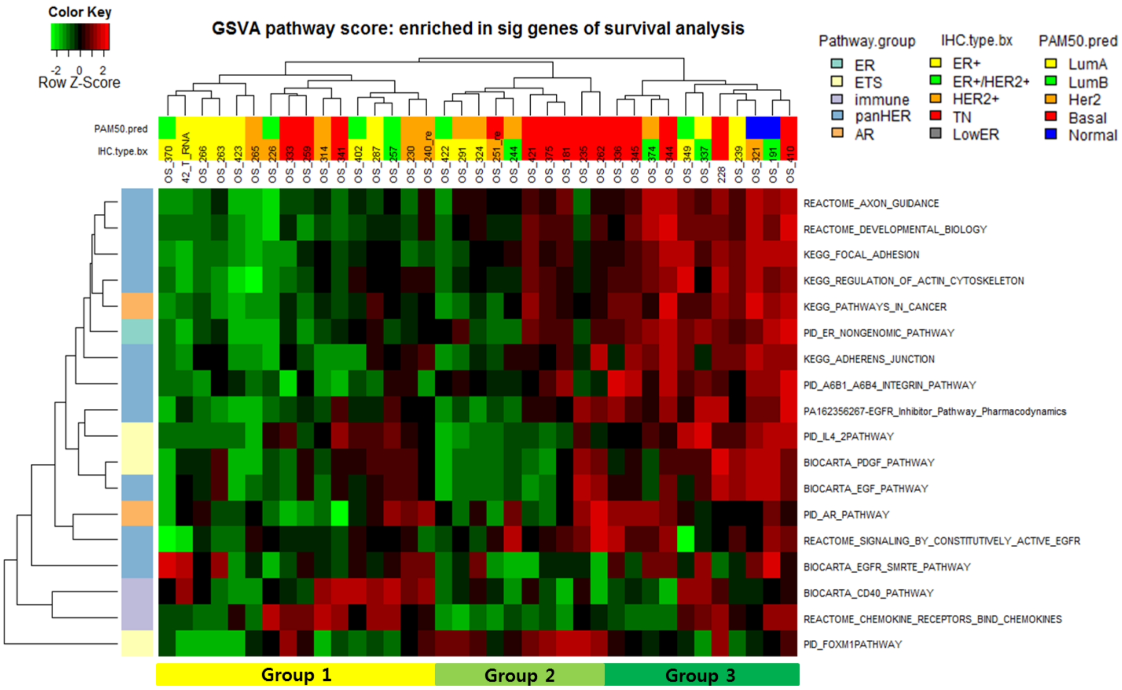

Supplementary Figure 8: Gene set variation analysis using 18 pathway gene sets.

Supplementary Table 1: Baseline characteristics according to AR expression (N=34)

	IHC type	Intrinsic subtype	AR RNA	AR	TP53	PIK3CA	BRCA1	BRCA2	ERBB2 amplification
1	ER+	LumA	up			p.E545K			
2	ER+	LumA	up			p.E674Q			
3	ER+	LumA	down						
4	ER+	LumA	up			p.E545K			
5	ER+	LumA	down		p.S176R	p.H1047R			
6	ER+	Her2	up						
7	ER+	Her2	down		-				
8	ER+	LumB	up			p.G1049R			
9	ER+	LumB	up					splicing	
10	ER+	LumB	up					p.Q3034X	
11	ER+/HER2+	Normal	up						
12	ER+/HER2+	Her2	up			p.H1047R			AMP
13	ER+/HER2+	LumB	up			p.H1047R			AMP
14	ER+/HER2+	LumA	down						AMP
15	ER+/HER2+	Her2	up			p.H1047R			AMP
16	HER2+	LumB	up		p.Y181C				
17	HER2+	Her2	down						
18	HER2+	Her2	up		p.R234H	p.H1047R			AMP
19	HER2+	Basal	up						AMP
20	HER2+	Her2	up		p.V235L				AMP
21	HER2+	Her2	up		p.L155R				AMP
22	HER2+	Normal	down						AMP
23	TN	Basal	down						
24	TN	Basal	down		p.R136H				
25	TN	Basal	down		p.Y181C				
26	TN	Basal	down	p.R102T		p.H1047R	splicing		
27	TN	Basal	down		p.E247V				
28	TN	Basal	down		p.C203G				
29	TN	Basal	down						
30	TN	Basal	down		p.R234H			p.T656S	
31	TN	Basal	down		p.R157X				
32	TN	Basal	down		-				
33	TN	Basal	down						
34	TN	Basal	down		p.R71P				

Supplementary Table 2: Genetic alterations according to AR expression (N=34)

Mutated gene	Mean. In group mutation	Mean. In group wildtype	Diff(mut-wild)	t.test fdr
ACACB	2.955	5.296	-2.341	0.21343
EVC	6.724	4.932	1.982	0.30411
GNAS	7.134	4.892	2.242	0.21343
LRRIQ1	7.109	4.820	2.289	0.12427
MUC16	7.201	4.808	2.393	0.01303
PCLO	2.921	5.300	-2.379	0.18242
PIK3CA	6.945	4.317	2.628	0.01694
TP53	4.446	6.270	-1.824	0.18242
TRIO	7.703	4.741	2.962	0.21343
TTN	6.623	4.761	1.862	0.18756
